# Zhen-Wu-Tang Protects IgA Nephropathy in Rats by Regulating Exosomes to Inhibit NF-κB/NLRP3 Pathway

**DOI:** 10.3389/fphar.2020.01080

**Published:** 2020-07-16

**Authors:** Honglian Li, Ruirui Lu, Yu Pang, Jicheng Li, Yiwen Cao, Hongxin Fu, Guoxing Fang, Qiuhe Chen, Bihao Liu, Junbiao Wu, Yuan Zhou, Jiuyao Zhou

**Affiliations:** ^1^ Department of Pharmacology, School of Pharmaceutical Sciences, Guangzhou University of Chinese Medicine, Guangzhou, China; ^2^ Department of Urology, The Sixth Affiliated Hospital of Sun Yat-sen University, Guangzhou, China; ^3^ Guangdong Institute of Gastroenterology, Sun Yat-sen University, Guangzhou, China; ^4^ The Second Affiliated Hospital, Guangzhou University of Chinese Medicine, Guangzhou, China

**Keywords:** Zhen-wu-tang, IgA nephropathy, exosomes, nuclear factor-κB, NLRP3 inflammasome

## Abstract

Immunoglobulin A nephropathy (IgAN) is one of the most frequent kinds of primary glomerulonephritis characterized by IgA immune complexes deposition and glomerular proliferation. Zhen-wu-tang (ZWT), a well-known traditional Chinese formula has been reported to ameliorate various kidney diseases. However, its pharmacological mechanism remains unclear. Exosomes have been described in diverse renal diseases by mediating cellular communication but rarely in the IgAN. The purpose of the present study is to explore whether the underlying mechanisms of the effect of ZWT on IgAN is correlated to exosomes. Our results demonstrated that in human renal tubular epithelial cells (HK-2) stimulated by lipopolysaccharide, exosomes are obviously released after ZWT-containing serum treatment especially with 10% ZWT. In addition, once released, HK-2-derived exosomes were uptaked by human mesangial cells (HMC), which impeded the activation of NF-κB/NLRP3 signaling pathway to exert anti-inflammatory effects in a lipopolysaccharide induced proliferation model. Moreover, IgAN rat model was established by bovine serum albumin, CCL_4_ mixed solution and LPS. We found that 10% ZWT could significantly promote the release of exosomes from HK-2 and inhibit HMC proliferation to improve inflammation. Thus HK-2-derived exosomes treated with 10% ZWT (ZWT-EXO) were administered to the rats by tail vein injection. Our results showed that ZWT-EXO decreased the levels of 24 h proteinuria, urinary erythrocyte, IgA deposition in glomerulus and renal pathological injury which ameliorated the kidney damage. In addition, ZWT was able to dramatically promote secretion of exosomes in renal tissues while blocked NF-κB nuclear translocation as well as activation of NLRP3 inflammasome, leading to the inhibition of IL-1β and caspase-1. In conclusion, our study reveal that ZWT has protective effects on IgAN by regulating exosomes secretion to inhibit the activation of NF-κB/NLRP3 pathway, thereby attenuating the renal dysfunction. These findings may provide a new therapeutic target for the treatment of IgAN.

## Introduction

IgA nephropathy (IgAN) is the most common type of primary glomerulonephritis, characterized by immune complex deposition, glomerular mesangial cell (GMC) proliferation and extracellular matrix (ECM) deposition (Coppo, 2018). The deposition of immune complex can activate mesangial cells, induce cytokines secretion and promote cell proliferation, resulting in inflammation and ultimately leading to kidney damage (Zachova et al., 2020). The main clinical manifestations of IgAN are macroscopic hematuria accompanied by proteinuria, severe hypertension and renal insufficiency. Accumulating evidences demonstrated that approximately 30%~40% of patients with IgAN developed into end-stage renal disease (ESRD) within 20 years (Moroni et al., 2019; Chen et al., 2020). Regrettably, the mechanism upon IgAN has not been fully understood, which impedes the design of the specific treatment for the disease. Therefore, it is essential to uncover appropriate drugs for the IgAN therapy.

Exosomes are small extracellular vesicles derived from endocytic membranes that can be secreted by nearly all body cells, they range in size from 30 to 150 nm (Dong et al., 2019). Exosomes are now considered as critical messengers in intercellular communications by transferring biomolecules such as RNA, lipids and enzymes to the recipient cells, and play an important role in cellular functions including inflammation and immune response. Given to these properties, they are recognized as promising biomarkers for diagnosis and prognosis of many diseases (Gurunathan et al., 2019). There is growing evidence that exosomes have therapeutic effects on the renal diseases, such as acute tubular injury (Pan et al., 2019) and chronic kidney disease (CKD) (Wang B. et al., 2019). Furthermore, it has been indicated that exosomes can improve renal ischemic-reperfusion injury (IRI)-induced renal structural injury by restraining nuclear factor-κB (NF-κB) activation to subside the expressions of inflammatory cytokines (Li L. et al., 2019). Nevertheless, the specific mechanism by which exosomes mediate inflammation in the IgAN is largely unclear.

NLRP3 inflammasome, a m\ultiprotein complex consists of NLRP3, ASC, and pro–caspase-1, is considered to be closely associated with the pathogenesis of IgAN (Tsai et al., 2017). Activation of NLRP3 inflammasome requires two steps namely priming and activation. Transcription of NF-κB is a priming step which encourages the expressions of NLRP3 and pro–IL-1β. After that, NLRP3 inflammasome activation occurs when exposed to something endogenous or another environmental stimulus. Consequently, caspase-1 is activated which in turns to initiate the secretion of IL-1β and IL-18 in an inflammatory condition (Zhang and Wang, 2019; Yu et al., 2020). NF-κB activation is deemed as a censorious move in the evolution of IgAN into ESRD (Zhang et al., 2017). Furthermore, NLRP3 gene expression is enhanced in biopsies of IgAN patients and highly localized to the tubular epithelium. The up-regulated level is relevant with renal tubular damage (Peng et al., 2019). Notably, previous studies have illustrated that activation of NF-κB/NLRP3 pathway plays a significant part in the IgAN by regulating inflammation, and it is efficacious to ameliorate IgAN by hampering this pathway to decrease the generation of downstream proinflammatory factors (Hua et al., 2013; Xiang et al., 2020). However, the relationship between exosomes and NF-κB/NLRP3 pathway in renal diseases particularly in IgAN remains concealed. Therefore, it’s indispensable to elaborate the specific mechanism upon exosomes and NF-κB/NLRP3 signaling pathway, and dig out efficient therapeutic drugs to recuperate IgAN.

More and more traditional Chinese medicine (TCM) has been used for prevention and treatment of IgAN and gradually approved worldwide (Li et al., 2017; Li and Li, 2020). Zhen-wu-tang (ZWT) is a classical prescription of *Treatise on Febrile Diseases* written by Zhongjing Zhang, composed of *Aconiti Lateralis Radix Praeparata, Poria, Atractylodis Macrocephalae Rhizoma, Paeoniae Radix Alba and Zingiberis Rhizoma Recens*. ZWT could protect different kidney diseases by possessing properties of anti-inflammation (Liu et al., 2016), antiapoptotic, (Liu et al., 2017), anti-oxidant (Li et al., 2018) and so on. Our previous study found that ZWT mitigated membranous nephropathy through depressing NF-κB pathway and NLRP3 inflammasome (Liu et al., 2019).In addition, ZWT decreased proteinuria, alleviated kidney pathology and podocyte damage *via* blocking NF-κB pathway in IgAN rats model (Liu et al., 2018). However, it is largely unclarified if ZWT protects against IgAN *via* regulating exosomes. Thus, in the current research, we hypothesized that ZWT protects IgAN in rats by inhibiting NF-κB/NLRP3 pathway by regulating exosomes. Our findings may provide new notions for the treatment of IgAN.

## Materials and Methods

### Preparation of ZWT

ZWT consists of five herbs (**Table 1**), which were purchased from Kangmei Pharmaceutical Co. LTD, Guangzhou, China (Lot. 190305041, 190250291, 190103501 and 190103501). The herbs were weighed accurately according to the clinical dose. After being soaked in eight times distilled water for 1 h, the ingredients were boiled to prepare ZWT as previously stated (Liang et al., 2014). The final aqueous extract was obtained with a concentration of 1.68 g raw materials per milliliter.

**Table 1:** The composition of Zhen-wu-tang (ZWT).

TCM materials (pinyin)	Latin name	Part used	Dry weight (g) of daily dose in clinic
Fuzi	*Aconitum carmichaelii* Debeaux	lateral radix	9
Fuling	*Poria cocos (Schw.)* Wolf	sclerotium	9
Baizhu	*Atractylodes macrocephala* Koidz	radix	6
Baishao	*Paeonia lactiflora* Pall	radix	9
Shengjiang	*Zingiber officinale* Roscoe	rhizome	9
Total			42

### HPLC Analysis of ZWT

High Performance Liquid Chromatography (HPLC) analysis was conducted to identify the main chemical components of ZWT. The concentration of ZWT was 1 g/ml (1 ml solution contains 1 g of the original herbs). The standards of paeoniflorin (Lot.S-010-180416), benzoylaconine (Lot.B-010-190814), benzoylmesaconine (Lot.B-016-180606), atractylenolide II (Lot.B-034-181216), atractylenolide III (Lot.B-034-171217), 6-gingerol (Lot.J-038-180515) and pachymic acid (Lot.F-006-191014) were provided by Chengdu Herbpurify CO., LTD (purity > 98%, Chengdu, China). HPLC was performed on Agilent 1260 Infinity (Agilent Technologies, California, USA) equipped with a Phenomenex LC column (250 ×4.6 mm, 4 µm). The mobile phase eluted with ultra-pure water (A) and methanol (B) in gradient mode. The proportion of methanol was varied from 5% ~ 80% ~5% in 95 min (0–5 min, 5%–5% B; 5–20 min, 5%–10% B; 20–45 min, 10%–20% B; 45–55 min, 20%–40% B; 55–70 min, 40%–60% B;70–75 min, 60%–80% B; 75–85 min, 80%–80% B; 85–90 min, 80%–5% B; 90–95 min, 5%–5% B) at a flow rate of 0.8 ml/min. The column temperature was 24°C and the detection wavelength was set at 230 nm.

### Drugs and Regents

Prednisone (Lot.1811231) was purchased from Suicheng Pharmaceutical Co. LTD, Zhengzhou, China. Bovine serum albumin (BSA, Lot.BSA190320) was obtained from Jiangsu Enmo Asai Biotechnology Co. LTD, China. Lipopolysaccharide (LPS, Lot.028M4094V) and GW4869 (Lot.0000063105) inhibitor of exosomes were provided by Sigma-Aldrich, USA. The ELISA kits for IL-1β (Lot.I17019960) and IL-18 (Lot.I29019961) were supplied by Cusabio Biotech Co. LTD, Wuhan, China. The antibodies goat anti-rabbit IgG/FITC (Lot. 120618), Rb a complement 3(C3, Lot. YE1110W), rabbit anti-rat IgA (Lot.13108) were from Bioss Biotechnology Co. LTD, Beijing, China. The antibody NLRP3/NLAP3 (cryo-2) mouse IgG2b (Lot. A27381510) was purchased from Life Science, USA. Anti-pro caspase-1 + p10 + p12 antibody (Lot.ab179515), rabbit anti-IL-1 beta antibody (Lot. ab9722), anti-CD9 antibody (Lot.ab92726), anti-CD81 antibody (Lot.ab109201), and anti-CD63 antibody (Lot.ab92726) were from Abcam, USA. HRP-linked anti-rabbit IgG (Lot.7074S), HRP-linked anti-mouse IgG (Lot.7076S),Alexa Fluor 488 anti-mouse IgG (Lot.4408S),Alexa Fluor 555 anti-mouse IgG (Lot.4409S), anti-p65 (Lot.8242) and anti-phospho-p65 (Ser536) (Lot.17) antibodies were provided by Cell Signaling Technology, USA. GAPDH mouse antibody (Lot.D2817) was purchased from Santa Cruz Biotechnology, USA. Exosomes-depleted fetal bovine serum (FBS) (Lot.180301-001) and ExoQuick-TC tissue culture media exosomes precipitation solution (Lot.180326-001) were purchased from System Biosciences, USA. The antibody ASC (Lot.2717) mouse monoclonal IgG1 was bought form Santa Cruz, USA. Dulbecco’s Modified Eagle Medium (DMEM)/F12, RPMI-1640 medium, fetal bovine serum, 100 U/ml penicillin and 100 μg/ml streptomycin were from Thermo fisher scientific, USA.

### Preparation of ZWT-Containing Serum

ZWT-containing serum (ZWTS) was manufactured as previously suggested (Liu et al., 2018). Concisely, fifty SD rats were randomly divided into two groups that one was treated with ZWT (16.8 g/kg), the other was treated with normal saline (10 ml/kg) to collect ZWTS and control serum (CS), respectively. Afterwards, blood samples were centrifuged at 845*g* for 10 min at 4°C. The serum was filtered after being deactivated at 56°C for 30 min, then stored at −20°C as a backup. Three concentrations of ZWTS were used in subsequent study: (1) 2.5% ZWTS with 7.5% CS as 2.5% ZWT (2) 5% ZWTS with 5% CS as 5% ZWT (3) 10% ZWTS as 10% ZWT.

### Cell Culture and Treatment

Human renal tubular epithelial cells (HK-2) were purchased from Kunming Cell Bank of the Chinese Academy of Sciences (Kunming, China). Human mesangial cells (HMC) were provided from analytical testing center of Central South University (Changsha, China). HK-2 cells were cultured in DMEM/F12 supplemented with 10% FBS, 100 U/ml penicillin and 100 μg/ml streptomycin. HMC were maintained in RPMI-1640 medium supplemented with 12% FBS, 100 U/ml penicillin, and 100 μg/ml streptomycin. HK-2 cells were treated with 2.5% ZWT, 5% ZWT, 10% ZWT, and GW4869 (40 μM) with or without LPS (100 ng/ml) for 24 h. Besides, HMC proliferation model was stimulated by LPS (20 µg/ml) for 24 h. A humidified atmosphere at 37°C supplemented with 5% CO_2_ was provided for cells incubation.

### Isolation of Exosomes

After being treated with different concentrations of ZWTS, cell culture supernatant from the HK-2 cells was acquired to isolate the exosomes. The supernatant was centrifuged at 4°C, 5,000*g* for 30 min to eliminate cell debris and then subjected to the exosomes ExoQuick-TC Tissue Culture Media Exosomes Precipitation Solution at 4°C overnight. The exosomes pellets (2.5% ZWT-EXO, 5% ZWT-EXO, 10% ZWT-EXO) were achieved after centrifugation for 30 min at 1500 g. BCA protein assay kits (Jiangsu, China) were used to determine the exosomes protein levels. The final concentration of ZWT-EXO for HMC was 16 µg/ml and 10 mg/ml for *in vivo* study.

### Transmission Electron Microscopy

The exosomes were dropped onto a carbon-coated electron microscopy grid and precipitated for 3 min. After being rinsed with PBS, the samples were stained with phosphotungstic acid and dried at room temperature for 5 min. The exosomes were identified by transmission electron microscope (JEM-1200EX, Japan).

### Nanoparticle Tracking Analysis

Nanoparticle tracking analysis (NTA) was performed with Nanosight ns300 (Malvern, UK). The exosomes were injected into the sample wells after diluted appropriately with PBS buffer. When the parameters were set, clicked “Creat and Run”, followed the on-screen prompts to complete the test. The particle size and concentration of the exosomes were obtained.

### Cell Proliferation Assay

Cells were seeded into 96-well plates and treated for 24 h at 37°C. After the indicated treatments, 10 μl MTT solution at 5 mg/ml (Biosharp, Guangzhou, China) was added into each well. One-hundred-fifty-microliter DMSO (Macklin, Shanghai, China) was applied to dissolve the MTT-formazan crystals after an additional culture at 37 °C for 4 h. Lastly, a microplate reader (Thermo Fisher Scientific, USA) was able to measure the absorbance at 450 nm.

### Co-Culture Experiment

HK-2 cells and HMC were cocultured by Transwell Permeable Support System of 12-well plates (0.4 µm pore-size,Corning, USA),.The former one was seeded in upper chamber while the recipient HMC were plated in the lower chambers. The cells were starved by serum-free medium for 12 h. Afterwards, HK-2 cells were labeled with 10 µM DiO dye (Beyotime, Shanghai, China) in the dark for 30 min. The cocultured experiment was carried out with or without ZWTS and GW4869 for 24 h. A confocal microscope (Zeiss, Germany) was managed to observe the uptake of DiO-labeled exosomes.

### Animals

Forty-eight SPF male Sprague-Dawley rats (Certificate No. SCXK 2018-0002) weighing 180~220 g were supplied by Guangdong Medical Laboratory Animal Center. The animal experimental procedures were conducted in accordance with the guidelines of the European Community and the National Institute of Health of the USA (NIH Publications No 8023, revised 1987), approved by the Animal Ethics Committee of Guangzhou University of Chinese Medicine (No.20181106002). All animals were housed at 25± 2°C and 65% humidity and provided for standard rat chows and tap water ad libitum. Except for 8 rats were selected randomly as a control group, the remaining rats were administered orally of BSA (600 mg/kg) every other day for 12 weeks, and 0.6 ml of castor oil (containing 0.1 ml of CCL_4_, Aladdin, Shanghai, China) was injected subcutaneously to per rat once a week for 12 weeks. At the same time, 0.3 ml of LPS solution was injected to per rat in caudal vein on the 6th, 8th, 10th week for once. Urine protein level for 24 h and microscopic hematuria were used as indicators of success in this rat model. After that, forty rats were randomly divided into model group, ZWT low-dose group (8.4 g/kg), ZWT high-dose group (16.8 g/kg), ZWT-EXO group (7.5 mg/kg), and prednisone group (2 mg/kg), and there were 8 rats in each group. Ten percent ZWT can obviously promote exosomes release from HK-2 and effectively inhibit HMC proliferation to improve inflammation. HK-2-derived exosomes treated with 10% ZWT were administered to the rats by tail vein injection as ZWT-EXO group. The others were administered intragastrically at the rat weight of 10 ml/kg with corresponding drugs. The control and the model groups were provided for equal volume of saline. The experiment lasted for sixteen weeks.

### Analysis of Urine Protein

No food for all animals but not prohibited from water on the 7th, 9th, 11th, 13th, 15th and 16th week, then the 24 h urine was collected to centrifuge at 3,500*g* for 5 min. Super-Bradford Protein Assay kits (CWBIO, Beijing, China) were used to detect the urine content.

### Blood Sampling and Tissue Removal

At the end of the experiment, animals were euthanized as previously introduced (Wu et al., 2018). The blood and renal tissue samples were obtained. The blood was centrifuged for 10 min at 1,400*g* to isolate the serum for further analysis. The kidney tissues were assigned into two parts. One part was fixed in 4% paraformaldehyde (Yongjin Biological Technology Co, LTD Guangzhou, China) for hematoxylin-eosin (HE) or Periodic Acid-Schiff (PAS) staining, and the other was quickly frozen in liquid nitrogen and maintained at −80°C as a backup for next detections.

### Serum Biochemical Analysis

The levels of blood urea nitrogen (BUN, Lot.20190709), serum creatinine (SCR, Lot.20190709), total protein (TP, Lot.20190711), albumin (ALB, Lot.20190712) were measured according to the instructions (Nanjing Jiancheng Bioengineering Institute, Nanjing, China).

### HE Staining

After being fixed in 4% paraformaldehyde, the renal tissues were dehydrated by gradient ethanol, and then embedded in paraffin. Subsequently, the tissues were cut into a 4 µm thickness and stained with hematoxylin-eosin. Lastly, the sections were visualized under a light microscope (OLYMPUS BX53, Shanghai, China) to evaluate histopathology.

### PAS Staining

The renal tissues were dehydrated and cut into 4-µm thick slices as mentioned in HE staining. Afterwards, the tissues were rinsed with ultrapure water after processing with periodic acid solution for 10 min. Then stained with schiff dye solution and washed successively with sodium sulfite solution and ultrapure water for 10 min. Subsequently, Mayer hematoxylin was used for counterstaining nucleus, alcohol hydrochloride for differentiation, and ultrapure water for rinsed. Finally, the tissues were assessed and taken images by the light microscope (OLYMPUS BX53, Shanghai, China).

### Immunofluorescence

The kidneys were embedded by optimal cutting temperature compound (OTC, Sakura, USA) and 6-μm sections were prepared with a freezing microtome (CryoStar NX50, Thermo Scientific, USA). The sections were fixed with pre-cooled acetone solution at 4°C for 15 min. After being washed with phosphate-buffered saline, the renal tissues were permeabilized with 0.5% Triton-X-100 (Dalian Meilun Biotechnology Co. LTD, Dalian, China). Next, the sections were blocked with 5% goat serum at room temperature for half an hour (Dalian Meilun Biotechnology Co. LTD, Dalian, China), incubated with the primary antibody IgA (dilution,1:200), IgG (dilution,1:200),C3 (dilution,1:200),CD63 (dilution,1:200), NLRP3 (dilution, 1:200) or ASC (dilution,1:200) at 4°C overnight protecting from light. The secondary antibody against IgG Fab2 Alexa Fluor 488 or 555 (dilution, 1:200) was employed to react with the sections, then stained with 4’,6-diamidino-2-phenylindole (Beijing Solibao Technology Co. Ltd, Beijing, China) for 5 min. Eventually, the immunofluorescence photographs were obtained by a laser confocal microscopy (Carl Zeiss, LSM800, Germany) after the samples were sealed with anti-fluorescent quencher (Dalian Meilun Biotechnology Co. Ltd, Dalian, China).

### ELISA Assay for IL-1β and IL-18

IL-1β and IL-18 levels in the renal tissues were quantified using the ELISA kits according to the manufacturer’s protocol. The absorbance was examined at 450 nm using the full-wavelength microplate reader (Thermo Fisher Scientific, USA) within 5 min.

### Western Blotting Analysis

Western blotting was fulfilled to measure the protein levels as previously mentioned (Wu et al., 2016). Protein samples of cells, renal tissues and exosomes were dissociated from SDS-PAGE, and transferred to PVDF membranes. After being subjected to 5% BSA (w/v) in TBST at room temperature, the membranes were treated with primary antibodies (1:1,000) overnight at 4°C. The primary antibodies were GAPDH, CD9, CD81, CD63, NLRP3, caspase-1 and pro–caspase-1, p-p65 NF-κB and p65 NF-κB, pro–IL-1β and IL-1β. After being rinsed thrice in TBST, the bands were incubated in goat anti-mouse or anti-rabbit secondary antibodies (1:5,000 or 1:10,000). An ECL kit (Millipore, MA, and USA) was required to visualize the reaction under an imaging system (Tanon 5200, Shanghai, China). Intensities of the bands were transformed by ImageJ 1.8.0 112 software. Protein expressions were normalized relative to appropriate controls.

### Statistical Analysis

SPSS 25.0 software (Chicago, IL, USA) was used for performing the statistical analysis. Data were expressed as mean ± standard deviation (SD). Comparisons of data among groups were carried out by one-way ANOVA, followed by Duncan’s test. *P*< 0.05 was considered statistically significant. Analyses were run by GraphPad Prism 8.0.

## Results

### HPLC Analysis of ZWT

**Figure 1:**
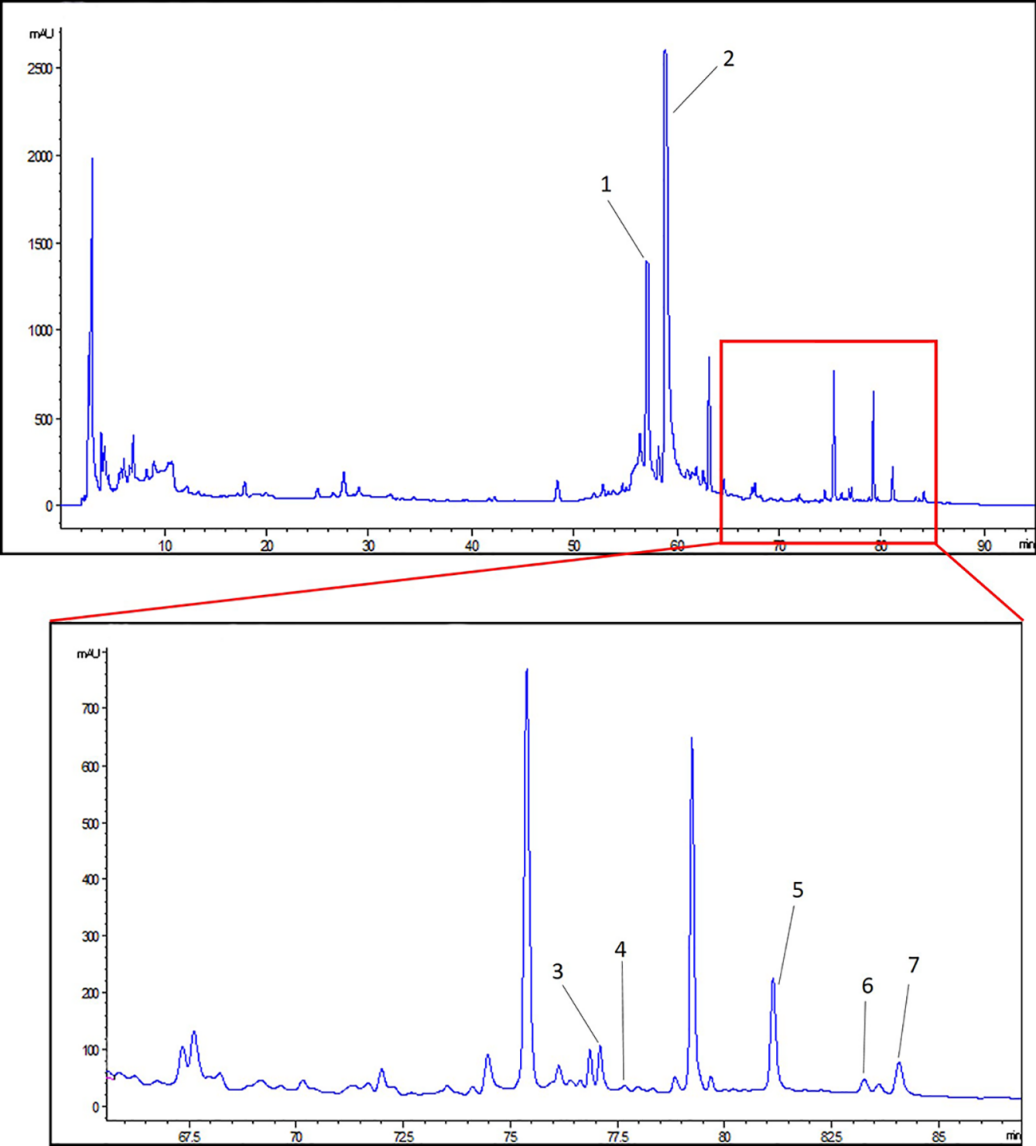
HPLC analysis of Zhen-wu-tang (ZWT). (1) 6-gingerol, (2) paeoniflorin, (3) benzoylaconine, (4) benzoylhypacoitine, (5) atractylenolide III, (6) pachymic acid, and (7) atractylenolide II.

The main components of ZWT was analyzed by HPLC method. A representative chromatogram of ZWT was shown in **Figure 1**. Among the components discovered in the extract of ZWT, a total of seven components were identified according to their retention times (**Table 2**).

**Table 2:** Herbal sources and retention times of seven components in Zhen-wu-tang (ZWT).

Constituents	Source	retention times (min)
6-gingerol	*Zingiber officinale* Roscoe	57.163
paeoniflorin	*Paeonia lactiflora* Pall	58.840
benzoylaconine	*Aconitum carmichaelii* Debeaux	77.102
benzoylhypacoitine	*Aconitum carmichaelii* Debeaux	77.670
atractylenolide III	*Atractylodes macrocephala* Koidz	81.140
pachymic acid	*Poria cocos (Schw.)* Wolf	83.269
atractylenolide II	Atractylodes macrocephala Koidz	84.080

### The Characterization of Exosomes and Intervention of ZWT

**Figure 2:**
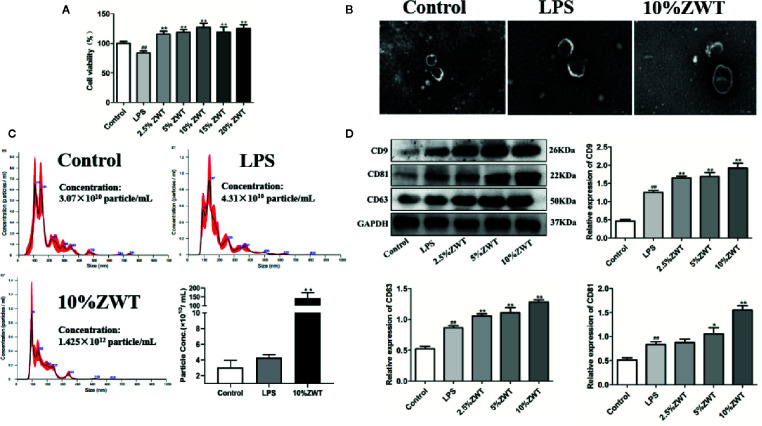
The characterization of exosomes and intervention of Zhen-wu-tang (ZWT). HK-2 cells were subjected to lipopolysaccharide (LPS) (100 ng/ml) and treated with or without ZWTS (2.5%, 5%, 10%, 15%, and 20%) for 24 h. **(A)** Cell vitality of HK-2 cells was examined by MTT assay. (n = 6). **(B)** Exosomes derived from HK-2 cells were identified by transmission electron microscopy (TEM). Magnification: ×15,000. Scale bar: 200 nm. (n = 3). **(C)** Size distribution of exosomes extracted from HK-2 cells conditioned medium by nanoparticle analysis (NTA). (n = 3). **(D)** Exosomal marker proteins including CD9, CD81, and CD63 in the supernatant of HK-2 cells were determined by western blot. (n = 3). The statistical data of all the proteins were analyzed and transformed by ImageJ 1.8.0 112 software. All values were shown as mean ± SD. *^#^P* < 0.05, *^##^P <* 0.01, compared with control group; ^*^P < 0.05, ^**^P < 0.01, compared with LPS group.

Studies have shown that HK-2 cells could regulate microenvironment and immune inflammatory response which play a significant role in the repair of renal injury (Hato et al., 2013). To assess this effect, we performed HK-2 cells-stimulated by LPS and intervention with ZWTS. The MTT assay showed that the cell viability of HK-2 cells was decreased by LPS, which was increased after intervention with ZWTS. In addition, 2.5% to 10% of ZWT had more significant protective effect on HK-2 cells, so this gradient concentration was used for subsequent experiments (**Figure 2A**). Exosomes were isolated from HK-2 cells and characterized *via* transmission electron microscopy (TEM) and NTA detection. TEM presented that all types of exosomes were classical round-shaped vesicles with double membrane structures (**Figure 2B**). There was a main peak of exosomal particle size of 109.0-, 136.3-, and 95.6-nm treated with control, LPS and 10% ZWT, respectively. Besides, the particle density was ascended a little bit when subjected to LPS (4.31×10^10^ particle/ml), but there was no remarkable difference compared with control group (3.07×10^10^ particle/ml). It was worth noting that the concentration of exosomes was spiked sharply when given to 10% ZWT (1.425×10^12^ particle/ml) (**Figure 2C**). Additionally, the expressions of exosomal protein markers were examined as well. As exhibited in **Figure 2D**, the expressions of CD9, CD81 and CD63 were promoted upon ZWTS intervention in comparison with LPS, and 10% ZWT treatment had the most significant promotion effect. These results indicated that ZWTS was able to stimulate HK-2 cells to secret exosomes.

### GW4869 Had a Negative Effect on the Release of Exosomes

**Figure 3:**
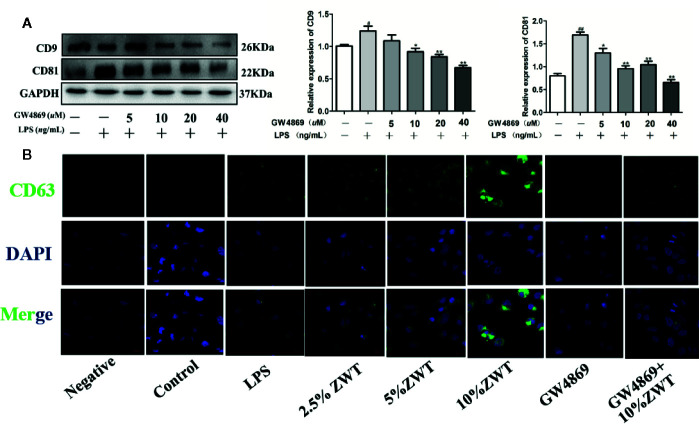
GW4869 had a negative effect on the release of exosomes. **(A)** Exosomal protein levels in the supernatant of HK-2 cells after GW4869 treatment were analyzed by western blot, 40 μM was the optimal concentration to inhibit the expressions of CD9 and CD81 (n = 3). The statistical data of all the proteins were analyzed and transformed by ImageJ 1.8.0 112 software. Data were expressed as mean ± SD. *^#^P* < 0.05, *^##^P <* 0.01, compared with control group; ^*^P < 0.05, ^**^P < 0.01, compared with lipopolysaccharide (LPS) group. **(B)** Confocal microscopic images of exosomes secreted from the HK-2 cells treated with or without LPS, ZWTS, and GW4869. Magnification: ×200. Scale bar: 20 μm.

At present, sphingomyelinase inhibitor GW4869 is basically an exosomes inhibitor recognized to impede exosomes liberation. To further confirm the impact of ZWT on exosomes production in HK-2 cells, GW4869 treatment was conducted to evaluate this effect. As seen from the western blot result, the addition of GW4869 at a concentration of 40 µM distinctly reduced CD9 and CD81 release. Therefore, the concentration of 40 µM was used for subsequent experiments (**Figure 3A**). Additionally, fluorescence intensity of CD63 increased in a dose-dependent manner after ZWTS therapy. In contrast, the staining intensity decreased after exposure to GW4869 (**Figure 3B**). It was revealed that GW4869 had a negative effect on exosomes release from HK-2 cells, hindering the effect of ZWT on exosomes liberation.

### ZWT Regulated Secretion of Exosomes, Which Affected NF-KB/NLRP3 Signaling Pathway in the HMC Proliferation Model

**Figure 4:**
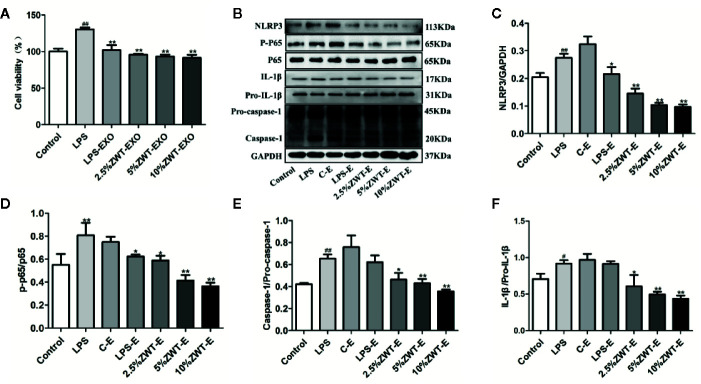
Zhen-wu-tang (ZWT) regulated secretion of exosomes, which affected NF-κB/NLRP3 signaling pathway in the human mesangial cell (HMC) proliferation model. HMC were treated with lipopolysaccharide (LPS) (20 μg/ml) and cultured in the absence/presence of ZWT-EXO for 24 h. **(A)** Cell proliferation of HMC by MTT analysis. (n = 6). **(B)** Western blot was carried out to measure the expressions of NLRP3, p-p65, p65, caspase-1, pro–caspase-1, IL-1β, and pro–IL-1β in the HMC. **(C–F)**The statistical data of all the proteins were analyzed and transformed by ImageJ 1.8.0 112 software. The obtained values of p-p65 were normalized to p65, caspase-1 were normalized to pro–caspase-1 and IL-1β were normalized to pro–IL-1β. (n = 3). All values were presented as mean ± SD. *^#^P* < 0.05, *^##^ P <* 0.01, compared with control group; ^*^P < 0.05, ^**^P < 0.01, compared with LPS group.

The pathogenesis of IgAN is complicated. Mesangial cell is activated when immune complexes come into glomerulus. After that, excessive cell proliferation and cytokines release trigger kidney injury (Sallustio et al., 2019). To elucidate the underlying protective mechanism of ZWT treatment in IgAN. An LPS-induced HMC proliferation model was designed *in vitro*. As ZWT could boost the exosomes secretion, we performed exosomes extraction from the HK-2 cells. Thus, whether ZWT ameliorating HMC proliferation through exosomes was studied. HMC proliferation was increased by LPS stimulation. This condition was attenuated after ZWT-EXO treatment and in the 10% ZWT-EXO group, the decrease was the most notable. (**Figure 4A**). Exosomes play a critical role in many kidney diseases through regulating NF-κB. NF-κB transcription urges NLRP3 activation which leads to the release of IL-18 and IL-1β. We then investigated whether ZWT-EXO regulated the NF-κB/NLRP3 signaling pathway during HMC proliferation. The results demonstrated that the expressions of p-p65, NLRP3, IL-1β and caspase-1 were up-regulated by LPS stimulation. After therapy of ZWT-EXO, the expressions of these proteins were significantly decreased (**Figures 4B**–**F**). These data displayed that ZWT regulated secretion of exosomes, which suppressed NF-κB/NLRP3 signaling pathway to lenify HMC proliferation.

### HK-2-Derived Exosomes Were Absorbed by HMC

**Figure 5:**
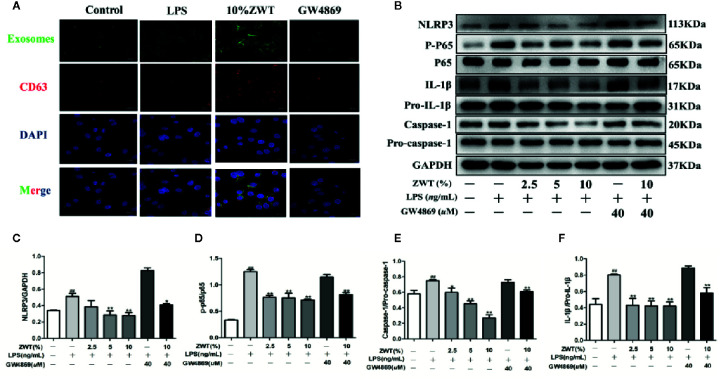
HK-2-derived exosomes were absorbed by HMC. **(A)** DiO-labeled HK-2 cells were cocultured with recipient cells of HMC using a transwell system for 24 h. **(B–F)** After being cocultured HK-2 cells and HMC, western blot was conducted to detect the expressions of NLRP3, p-p65, p65, caspase-1, pro–caspase-1, IL-1β and pro–IL-1β in the HMC. The statistical data of all the proteins were analyzed and transformed by ImageJ 1.8.0 112 software. The obtained values of p-p65 were normalized to p65, caspase-1 were normalized to pro–caspase-1 and IL-1β were normalized to pro–IL-1β. (n = 3). All values were presented as mean ± SD. *^#^P* < 0.05, *^##^ P <* 0.01, compared with control group; ^*^P < 0.05, ^**^P < 0.01, compared with LPS group.

When exosomes are released, the next step is the uptake by recipient cells (H et al., 2017). In order to explore the communication between HK-2 and HMC, we established a coculture system. A 0.4-μm transwell filter was applied to prevent the DiO-labeled HK-2 and HMC contact directly. As expected, the fluorescence-labeled exosomes were observed in the HMC, and the uptake ability was reinforced in the group of ZWTS-treated while GW4869 also exerted an opposite side (**Figure 5A**). To clarify this internalization of exosomes inside HMC whether influence the NF-κB/NLRP3 pathway, we next performed the protein assay. After being cocultured, the expressions of p-p65, NLRP3, IL-1β, and caspase-1 in the HMC were enhanced by LPS. This condition was markedly reversed by treatment of ZWTS especially with 10% ZWT. In addition, when the exosomes were blocked by GW4869 treatment, the effect of ZWTS was weaken (**Figures 5B–F**). As a result, exosomes could be taken in by HMC, and down-regulation of NF-κB/NLRP3 pathway was associated with increased uptake of exosomes after ZWT treatment.

### ZWT-EXO Improved Renal Function of IgAN Rats

**Figure 6:**
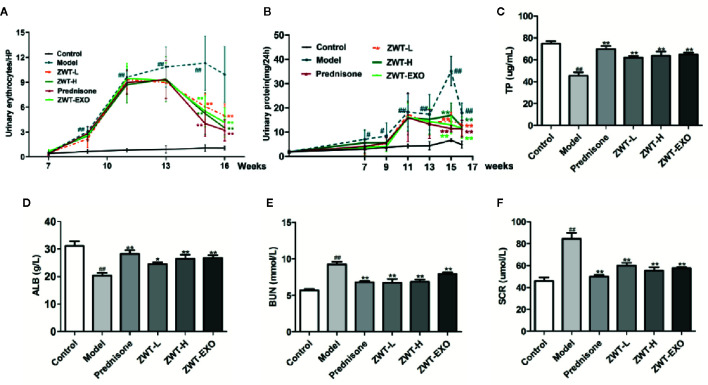
ZWT-EXO improved renal function of immunoglobulin A nephropathy (IgAN) rats. **(A)** 24-h urine protein of the IgAN rats in the different periods. (n = 8). **(B)** Urinary erythrocyte numbers at high power field of the IgAN rats in the different periods. (n = 8). **(C–F)** The content of total protein (TP), albumin (ALB), blood urea nitrogen (BUN), and serum creatinine (SCR) in the serum of IgAN rats (n= 5). All values were presented as mean ± SD. *^#^P* < 0.05, *^##^ P <* 0.01, compared with control group; ^*^P < 0.05, ^**^P < 0.01, compared with lipopolysaccharide (LPS) group.

To investigate the impact of exosomes on the IgAN, we set up an IgAN rat model *in vivo*. As 10% ZWT treatment could further facilitate exosomes release form HK-2 and more effectively suppressed inflammation in the HMC proliferation model. Exosomes were isolated from HK-2 cells treated with 10% ZWT, the rats were administered by tail vein injection as ZWT-EXO group. ZWT-EXO was able to reduce the 24-h urine protein (**Figure 6A**) and the urinary erythrocyte numbers (**Figure 6B**) compared with the model group. TP, ALB, BUN and SCR indicators were also detected to assess the renoprotective effects of ZWT-EXO. Compared with the model group, content of TP (**Figure 6C**) and ALB (**Figure 6D**) were upregulated while the BUN (**Figure 6E**) and SCR levels (**Figure 6F**) were declined after undergoing ZWT-EXO injection. These data suggested that ZWT-EXO played a protective role in IgAN rats through improving renal function.

### ZWT-EXO Dampened Renal Inflammation and Structure Damage of IgAN Rats

**Figure 7:**
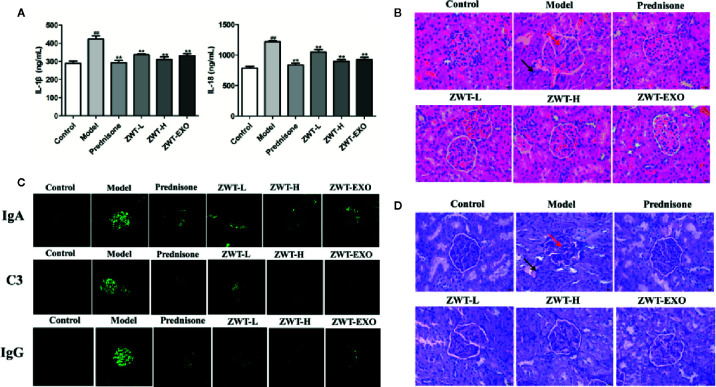
Exosomes dampened renal inflammation and structure damage of immunoglobulin A nephropathy (IgAN) rats. **(A)** IL-1β and IL-18 levels in the IgAN renal tissues from the different groups. (n= 5). **(B)** The photographs of H&E staining in IgAN renal tissues under a light microscope at 400× magnification. Scale bar: 20 μm. The arrows indicated the partial pathological changes as inflammatory infiltration with black arrow and glomerulus swelling with red arrow. **(C)** Circulating immune complexes of IgA, IgG, and C3 in the renal tissues were examined by confocal imaging at ×200 magnification. Scale bar: 20 μm. **(D)** The images of IgAN renal tissues by Periodic Acid-Schiff (PAS) staining under a light microscope at ×400 magnification. Scale bar: 20 μm. Black arrow: mesangial cells proliferation and cellular infiltration. Red arrow: matrix dilation.

Renal functional decline of IgAN is related to the expression of inflammatory changes (Deng et al., 2019). We undertook ELISA assay to determine the inflammatory content of IL-1β and IL-18. As shown in the **Figure 7A**, the expressions of IL-1β and IL-18 were apparently raised in the model group compared with the normal one. Significant lower levels of these inflammatory cytokines displayed in the ZWT-EXO group. Kidney sections were subjected to PAS staining and HE staining to evaluate the renal histology. The IgAN kidneys displayed abnormal morphology, structural disorder and glomerulus swelling. The obvious mesangial cells proliferation in untreated rats was accompanied by cellular infiltration and matrix dilation. These pathological changes were limited in rats administered with ZWT-EXO (**Figures 7B, D** **)**. IgAN is mainly characterized by immune complexes deposition in the mesangial area, evoking activation of the complement system such as C3.We found that there were conspicuous fluorescence signals of IgA and IgG in the IgAN kidneys. Meanwhile, the C3 complement was expressed higher than that in the control group. On the contrary, slight signals were observed in rats infused with ZWT-EXO (**Figure 7C**). These results indicated that ZWT-EXO could alleviate renal inflammation and pathological damage *via* dramatically dampening the deposition of immune complexes in IgAN rats.

### ZWT Was Able to Reinforce the Secretion of Exosomes in IgAN Rats

**Figure 8:**
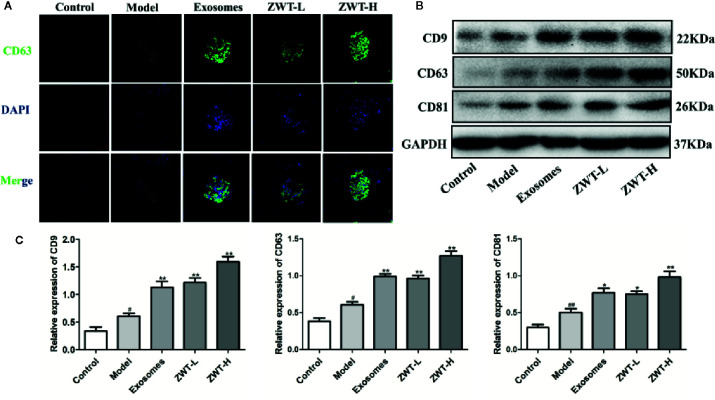
Zhen-wu-tang (ZWT) was able to reinforce the secretion of exosomes in immunoglobulin A nephropathy (IgAN) rats. **(A)** CD63 expression in the glomerulus was evaluated by confocal imaging at ×200 magnification. Scale bar: 20 μm. **(B, C)** Western blotting of CD9, CD63, and CD81 expressed in the glomerulus of IgAN rats. (n= 3).All values were presented as mean ± SD. *^#^P* < 0.05, *^##^P <* 0.01, compared with control group; ^*^P < 0.05, ^**^P < 0.01, compared with LPS group.

*In vitro* study, we have demonstrated that the expression of exosomes in the HK-2 cells is significantly upregulated by the intervention of ZWTS. To further confirm the underlying mechanism of ZWT on IgAN rats, we had new findings. We unearthed that CD63 was hardly presented in the normal rats but distributed stronger in the IgAN glomerulus. After intervention of ZWT, CD63 fluorescence was further strengthened especially in the high-dose group, as well as in the ZWT-EXO group (**Figure 8A**). Similar result was discovered in the expression of CD63 protein (**Figures 8B, C** **)**. Moreover, CD81 and CD9 levels in the renal tissues were also notably intensified in the ZWT-treated rats (**Figures 8B, C** **)**. These results demonstrated that ZWT could plainly facilitate exosomes release of kidney in IgAN rats.

### ZWT Blocked the NF-κB/NLRP3 Signaling Pathway in IgAN Model Rats

**Figure 9:**
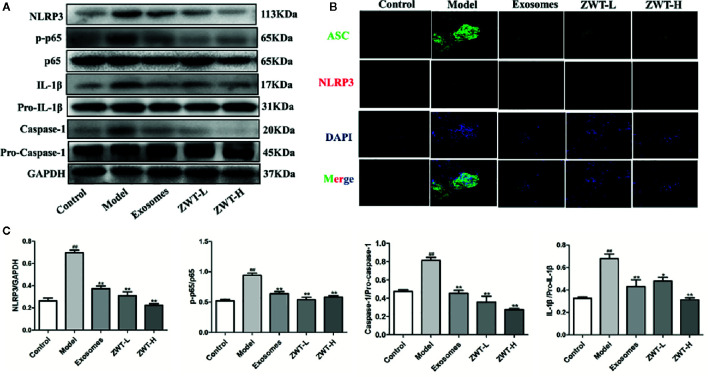
ZWT blocked the NF-κB/NLRP3 signaling pathway in immunoglobulin A nephropathy (IgAN) model rats. **(A, C)** Western blot was carried out to investigate the expressions of NLRP3, p-p65, p65, caspase-1, pro–caspase-1, IL-1β, and pro–IL-1β in the kidney tissues of IgAN rats. The statistical data of all the proteins were analyzed and transformed by ImageJ 1.8.0 112 software. The obtained values of p-p65 were normalized to p65, caspase-1 were normalized to pro–caspase-1 and IL-1β were normalized to pro–IL-1β. (n= 3). Data were shown as mean ± SD. *^#^P* < 0.05, *^##^P <* 0.01, compared with control group; ^*^P < 0.05, ^**^P < 0.01, compared with lipopolysaccharide (LPS) group. **(B)** The colocalization images of NLRP3 and ASC were measured by immunofluorescence with confocal imaging at ×400magnification. Scale bar: 20 μm.

It was reported that NF-κB-activated immunoreactive inflammation was a hinge step in the development of IgAN into ESRD (Zhang et al., 2020). Translocation of NF-κB would empower the expressions of NLRP3 and pro–IL-1β. Subsequently, NLRP3 recruited ASC and pro–caspase-1 proteins. The inflammasome was formed to encourage the active expressions of IL-1β and IL-18, which finally incited phlogistic response *in vivo* (Ren et al., 2019). As seen in the results, the protein levels of p-p65, NLRP3, caspase-1 and IL-1β were increased in the renal tissues of IgAN rats. In contrast, these levels were significantly repressed by ZWT-EXO and ZWT (**Figures 9A, C** **)**. Otherwise, colocalization of ASC and NLRP3 in the kidney tissues was highly expressed in the IgAN rats compared with the control group. Luckily, the condition was curbed after following ZWT-EXO or ZWT treatment (**Figure 9B**). Taken together, these data suggested that ZWT inhibited the NF-κB/NLRP3 signaling pathway in IgAN model rats, and ameliorated inflammation response *via* exosomes.

## Discussion

ZWT is a traditional Chinese herbal prescription that has been widely applied to remedy many kidney diseases for years in China. In the previous study, we have proved that ZWT showed a good improvement effect on IgAN (Liu et al., 2018). However, the accurate pathogenetic mechanisms have not been fully acknowledged. Here, HK-2-derived exosomes treated with ZWT were isolated to treat IgAN for the first time and demonstrated that they could relieve renal damage of IgAN rats by suppressing HMC proliferation and down regulating NF-kB/NLRP3 pathway.

Exosomes are widely distributed in various body fluids, which carry diverse biological content including DNA, RNA and protein (He et al., 2018). When the exosomes are isolated, it is necessary to identify the morphological structure and particle size. Some standard methods have been used to identify exosomes. The size and shape can be characterized by NTA and TEM (Wang et al., 2016). Besides, exosomes membranes are enriched in tetraspanins such as CD9, CD63 and CD81 which are thought to play a key role in exosomes biogenesis, and are widely used as exosomes markers. They can be examined by western blot and flow cytometry (Whiteside, 2016). In our work, the results of TEM showed that exosomes were cup-shaped extracellular microvesicles with a lipid bilayer membrane structure. From the analysis of NTA, we discovered that the main peaks of the particle sizes in the control, LPS and ZWT group were 109.0, 136.3, and 95.6 nm, respectively. They were in the range of 30 to 150 nm in accordance with the NTA standard. The tetraspanins including CD9, CD81, and CD63 are also clearly observed through western blotting data. Namely, we isolated the exosomes successfully.

IgAN is a common type of chronic glomerular diseases worldwide (Luan et al., 2019). Multiple factors are involved in the pathogenesis of IgAN. When circulating complexes deposit in the glomeruli, the GMCs are activated, followed by cell proliferation and excessive production of inflammatory factors that promote impaired renal function (Chang and Li, 2020). As a new kind of biological marker, exosomes can not only mediate intercellular communication in inflammation but also remove waste or harmful components, which participate in the development of many renal deseases (Li Q. et al., 2019). Particularly, a new report illustrated the circRNAs carried by exosomes from HG-treated glomerular endothelial cells (GECs) activate GMCs, thus promoting kidney fibrosis in diabetic nephropathy. (Ling et al., 2019). The interaction between HK-2 and HMC has been considered closely related to the pathogenesis of IgAN. However, the detailed relationship between them in the development of IgAN has not been investigated. Hence, we performed this study to articulate their connection in IgAN. We found that exosomes secretion was extraordinarily intensified from HK-2 cells after undergoing ZWT treatment. HMC proliferation was muffled by the ZWT-EXO especially with the 10% ZWT. We injected 10% ZWT-EXO into the rats, and the influence of ZWT-EXO on IgAN was evaluated. The results indicated that ZWT-EXO intervention obviously decreased hematuria and proteinuria. And the pathological injuries were also improved with reduction of accumulated immune complexes which restored the renal function.

NF-κB is discovered as a crucial mediator of inflammation and immunity, which involved in pathophysiological processes such as generation of cytokines and chemokines (Mussbacher et al., 2019). Several previous studies showed that NF-κB played a significant role in the processes of IgAN (Zhang and Sun, 2015; Yuan et al., 2017). NF-κB expression is negatively correlated with the prognosis of IgAN patients (Silva et al., 2011). Besides, there is growing evidence that exosomes can mitigate various inflammatory responses by inhibiting the NF-κB signaling pathway (Duan et al., 2019; Wang Z. et al., 2019). However, it is rarely reported in IgAN. In this work, NF-κB was activated in the LPS stimulated HMC proliferation model and IgAN rats. Interestingly, it was curbed after undergoing ZWT-EXO treatment. In addition, ZWT treatment accelerated the expressions of exosomal markers in the kidney tissues, which made us further convince that ZWT was beneficial for the treatment of IgAN *via* regulating exosomes.

NLRP3 inflammasome has been increasingly focused due to aggravating the inflammation in diverse renal diseases (Fan et al., 2019; Seo et al., 2019). It was described that NLRP3 mRNA in the serum of IgAN patients was higher than the healthy people. In addition, NLRP3 took part in the pathogenesis of IgAN in an inflammasome-dependent way (Kim et al., 2019). It is well known that transcription of NF-κB is a priming step of NLRP3 inflammasome activation. Afterwards, caspase-1 is matured by cleavage of pro–caspase-1, which pushes IL-1β and caspase-1 to be matured. As a result, inflammatory response is sparked off which participates in the occurrence of immune inflammatory diseases (Lu et al., 2019). However, there is little information about the molecular mechanisms between exosomes and NLRP3 inflammasome. It is shown that once exosomes are released, the next step is the uptake of their contents by the recipient cells, and some exosomes-induced cellular responses do not require the recipient cells to absorb the exosomes. Signal cellular communication can be caused by adhesion of exosomes surface proteins to receptors expressed on the recipient cells (Dassler-Plenker et al., 2020). Therefore, HK-2-derived exosomes were extracted and applied to the HMC proliferation model to observe the direct effect. Meanwhile, a coculture system was also performed to investigate the active uptake ability of HMC. In the present study, we found that ZWT-EXO could directly suppressed NF-κB/NLRP3 signaling pathway to mitigate HMC proliferation. Furthermore, HK-2-derived exosomes could be internalized into HMC, and ZWT promoted this uptake effect and reduced the protein levels of NLRP3, IL-1β, and caspase-1.However, the uptake effect was weakened when subjected to GW4869 intervention which had an impact on the expression of the NF-κB/NLRP3 signaling pathway. Besides, the colocalization of ASC and NLRP3 in the renal tissues was curbed after ZWT or ZWT-EXO treatment. Therefore, ZWT-EXO plays an important role in regulating NF-κB/NLRP3 pathway to relieve IgAN.

It is noted that our work has some limitations and should be elaborated in the future research. Firstly, exosomes can also carry biological genetic components such as mRNA and microRNA. Thus additional studies are necessary to clarify what kind of specific content is playing a role. Secondly, the location of the exosomes distributed in the target organs should be confirmed in IgAN model rats. Finally, ZWT consists of five traditional Chinese medicines with complex ingredients. It is essential to further clarify the effective compounds of ZWT on IgAN treatment and the precise targets.

In conclusion, our results provide new evidence that ZWT has protective effects on IgAN by regulating exosomes secretion. NF-κB/NLRP3 pathway is involved in the onset and progression of IgAN rats. Strengthening the secretion of exosomes to restrain NF-κB/NLRP3 signaling pathway is a prospective mechanism of ZWT treatment on IgAN. Our findings may provide new notions for the treatment of IgAN. Maybe we can try to explore new drugs from the perspective of exosomes in the future of clinical trials.

## Data Availability Statement

All datasets presented in this study are included in the article/supplementary material.

## Ethics Statement

The animal study was reviewed and approved by Animal Ethics Committee of Guangzhou University of Chinese Medicine.

## Author Contributions 

JZ and YZ conceived the experiments. HL, RL, YP, and JL conducted the *vivo* research. YC, HF, and GF carried out the *vitro* research. QC, BL, and JW contributed to analyse the data. HL, JZ and YZ wrote the manuscript. All authors participated in the discussion of the study.

## Funding

This research was supported by the National Natural Science Foundation of China (Grant No.81673874, 81803824 and 81603371), and the Natural Science Foundation of Guangdong Province China (Grant No. 2018A030313328 and 2018B0303110004).

## Conflict of Interest

The authors declare that the research was conducted in the absence of any commercial or financial relationships that could be construed as a potential conflict of interest.
